# Physical activity combined with sedentary behaviour in the risk of mortality in older adults

**DOI:** 10.11606/s1518-8787.2021055003461

**Published:** 2021-10-13

**Authors:** Lucas Lima Galvão, Rizia Rocha Silva, Sheilla Tribess, Douglas Assis Teles Santos, Jair Sindra Virtuoso

**Affiliations:** I Universidade Federal do Triângulo Mineiro Programa de Pós-Graduação em Educação Física UberabaMG Brasil Universidade Federal do Triângulo Mineiro. Programa de Pós-Graduação em Educação Física. Uberaba, Minas Gerais, MG, Brasil; II Universidade Federal do Triângulo Mineiro Instituto de Ciências da Saúde Departamento de Ciências do Esporte UberabaMG Brasil Universidade Federal do Triângulo Mineiro. Instituto de Ciências da Saúde. Departamento de Ciências do Esporte. Uberaba, Minas Gerais, MG, Brasil; III Universidade do Estado da Bahia Departamento de Educação Teixeira de FreitasBA Brasil Universidade do Estado da Bahia. Departamento de Educação. Teixeira de Freitas, Bahia, BA, Brasil

**Keywords:** Aged, Sedentary Behavior, Exercise, Risk Factors, Mortality

## Abstract

**OBJECTIVE:**

To examine the effects of physical activity (PA) and sedentary behaviour (SB), in isolation and combination, on all-cause mortality in older adults.

**METHODS:**

Prospective, population-based cohort study. The data were collected from first wave in 2015 and the follow-up continued until 2020. The sample consisted of 332 older adult people aged ≥ 60 years-old, out of which 59 died. The level of PA and SB was assessed by the International Physical Activity Questionnaire (IPAQ). The older adults were divided into PA categorized as sufficiently active and insufficiently active and into high and low SB. We built four combinations of PA and SB. Also, we used the Cox proportional hazards regression with a 95% confidence interval with hazard ratio estimate so as to verify the mortality risks between PA, SB, and the combinations of PA and SB.

**RESULTS:**

Insufficiently active individuals had higher risks of mortality compared to sufficiently active people. We observed no associations between SB and mortality separately; however, when evaluated in a combined way, insufficiently active individuals and with a high SB time had a higher chance of mortality compared to active individuals with a low SB time.

**CONCLUSION:**

Our isolated analyses demonstrate that complying with PA recommendations reduces the risk of mortality; however, no association was found between the time of PA exposure with the time of SB. When analysing the combination, being physically inactive and with a long time of SB showed higher mortality rates, with SB being an enhancer of this risk. The results of this study show the interdependence of SB for PA performed at moderate to vigorous intensity. The understanding of this interrelation must be considered in the formulation of public health guidelines.

## INTRODUCTION

Regular physical activity (PA) is considered a key determinant of global longevity^1^, being constantly associated with a reduced risk of chronic non-communicable diseases (NCDs), such as cardiovascular disease^2^, diabetes mellitus^3^, some types of cancer^4^, showing how severe the mortality^5,6^ is. Currently, PA recommendations for the older adult population describe that individuals should accumulate between 150 to 300 minutes of moderate-intensity PA weekly, or 75 to 150 minutes of vigorous PA, or a combination of both, in addition to participating in multicomponent activities, such as balance training, aerobic activities and muscle-strengthening^7^. However, some studies have found positive results with PA performed at lower intensities^5,6^, where the increase in PA of mild intensity was associated with a 17% lower risk of mortality^8^.

The term “sedentary behaviour” (SB) establishes an opposite to PA, being associated with several health problems^9,10^, including mortality^11^. SB is conceptualized as activities performed in a sitting, lying, or reclining position with energy expenditure ≤ 1.5 metabolic equivalents (METs)^12^. The long-term exposure to SB can be harmful to health, regardless of the level of PA^10,13^. This idea is refuted by others^14,15^, who point out that PA is a determining factor for positive health outcomes. As observed in a meta-analysis including 16 studies, with follow-up of more than one million individuals, the association between SB was found and the risk of mortality was gradually attenuated with increases in the time spent in moderate-intensity PA of 60 to 75 minutes a day^16^. These values may be higher than the recommendations for PA.

SB presents independent effects on PA on human metabolism, physical function, and health outcomes^17^. Likewise, the studies found in the literature point to a greater tendency for the independent effect of SB and PA on mortality, however, such studies are performed, based on samples with a wide age range and lack of sound evidence^18^.

Due to the lack of prospective studies on the mutual effects of PA and SB on all-cause mortality, understanding the combined associations between PA and SB is of paramount importance for the development of public guidelines^15,19^. Considering that the day has24 hours and behaviours may vary, studying the behaviour patterns about PA and SB is important.

Thus, this study aimed to examine the effects of PA and SB, in isolation and combined, on all-cause mortality in older adults.

## METHODS

### Study Design and Participants

As part of the *Estudo Longitudinal de Saúde do Idoso de Alcobaça* (ELSIA – Longitudinal Health Study for the Older Adults of Alcobaça), the household survey, observational and with a longitudinal design, was conducted with individuals aged 60 years old or over, living in an urban area and registered in the program Estratégia Saúde da Família (ESF – Family Health Strategy) from the municipality of Alcobaça, Bahia, Brazil. Baseline data collection took place from July to October 2015, and follow-up, from January to February 2020.

The exclusion criteria were as follows: being bedridden; being hospitalized; being a resident of long-term institutions; having great difficulty in visual and auditory acuity that can hinder communication with the interviewer; being dependent on a wheelchair; having musculoskeletal or neurological diseases that prevent the measurement of physical function; scoring less than13 points in the Mini-Mental State Examination (MMSE)^20^, and not agreeing to participate in the study.

For the baseline, 54 of the 743 individuals registered in the FHS refused to participate in the research, 58 were excluded based on the study criteria (six were wheelchair users; 10 were bedridden; 19 had a previous diagnosis of diseases or dysfunctions that prevented the interviews; 14 had MMSE, eight had communication difficulties and one was an alcoholic), 158-older adults could not be contacted after three attempts and 54 did not have data for all the specific variables of the present study. Thus, the data of 419 older adults were analyzed in this study.

Data collection took place with the help of community health agents in the municipality, eligible older adults were located and informed about the research objectives. Those who agreed to participate signed an informed consent form, participated in a face-to-face interview to obtain sociodemographic information (age, sex, school years, home arrangement, and income), health and behaviour, and underwent examinations, such as physical function tests. Interviewers were previously trained.

For the second wave of data collection, we used geographic information (latitudes and longitudes), and addresses obtained in the first wave of the collection to locate individuals. If this contact was not possible, we sought the older adults through information from neighbors, relatives, personal or family phones made available in the first wave of collection by the participants. Of those included in the first study, 105 were not found and 36 moved to another city, totaling 141 segment losses; of the 332 statements obtained, 59 died and 273 were alive.

### Ethical Procedures

The study protocol and procedures were carried out following the Declaration of Helsinki and were previously approved by the Human Research Ethics Committee of the Federal University of *Triângulo Mineiro* (Ordinance No. 966.983/2015) and the State University of Bahia (Ordinance No. 3,471,114/2020).

We invited the individuals to participate and share information about the nature and objectives of the research. After the interviewee’s consent and signing the Informed Consent Form, we conducted the study.

### Mortality

Participants were followed up until the moment of death, loss of follow-up or until end of the second wave (February 29, 2020). The vital status was determined through telephone follow-up, information from family members with presentation of the death certificate, information obtained from the municipal registry, and/or consultation on the website of the Court of Justice of the State of Bahia. For this study, we included mortality from all causes, and we calculated the follow-up time from the beginning of the survey to death.

### Physical Activity and Sedentary Behaviour

The level of PA and the time exposed to SB were assessed using the international PA questionnaire, long version^21^, validated to assess the level of PA and SB^22^, including in the older adult Brazilian population^23,24^. The level of PA was determined from activities of moderate to vigorous intensity (MVPA) performed for at least 10 continuous minutes, assessed in the PA domains of leisure-time, work, transportation and household tasks. The variable was divided into sufficiently active (≥ 150 min/week of moderate physical activity or ≥ 75 min/week of vigorous intensity physical activity or a combination of both) and insufficiently active (< 150 min/week of moderate physical activity or < 75 min/week of vigorous intensity physical activity)^19^.

SB was determined by the time spent sitting, assessed from the questions of sitting time on a typical workday (‘How much time do you spend sitting in a workday?’) and a regular weekend day (‘How much time do you spend sitting in a weekend day?’). We determined the total sitting time, measured as the number of minutes per day from the weighted average of sitting time on a workday and weekend day: (sitting time on a weekday × 5 + sitting time on a weekend day× 2)/7). The SB time was considered elevated from the 75th percentile (P75 = 540 minutes/day).

### Combinations

The combinations were built jointly between the PA and SB levels, resulting in four groups with varied behaviours, from the best behaviour (≥ 150 min/week and < P75 min/day), intermediate behaviours (≥ 150 min/week and ≥ P75 min/day; < 150 min/week and < P75 min/day) and to the worst behaviour (< 150 min/week and ≥ P75 min/day).

### Covariables

We evaluated the covariables in the first wave of the study, comprising: gender (male and female); home arrangement (with a partner and without a partner); self-reported health status (positive or negative); smoking (yes or no); age (continuous years) and the number of diseases (continuous numbers).

We obtained the body mass index (BMI) in the equation body mass/height^2^. We obtained the body masses of participants using a portable digital scale (brand: Welmy; model: W-200M), which had a ranging of 0.1 kg and a capacity of up to 200 kg. The participants were weighed only once in the standard anatomical position, without shoes and wearing light clothing. We obtained the height of participants using a portable stadiometer (brand: WCS; model: Compact) that measured a range between 0 and 220 cm. Height was measured while the participants held their breath.

Participants did not wear shoes, maintaining their heels together in the standard anatomical position, and were instructed to position their heads according to the Frankfurt plane.

The measurements were taken at the participant’s residence by previously trained examiners (academics and health professionals).

### Data Analysis

We entered data in duplicate in the Epidata software (version 3.1b), and all statistical analyses used the SPSS software (version 23.0). To test the normality of the data, we used the Kolmogorov–Smirnov. Descriptive statistics with dispersion calculations, absolute and relative frequencies characterized the sample. The chi-square test (qualitative variables) and Mann–Whitney U test (quantitative variables) compared vital status and descriptive variables.

**Figure 1:**
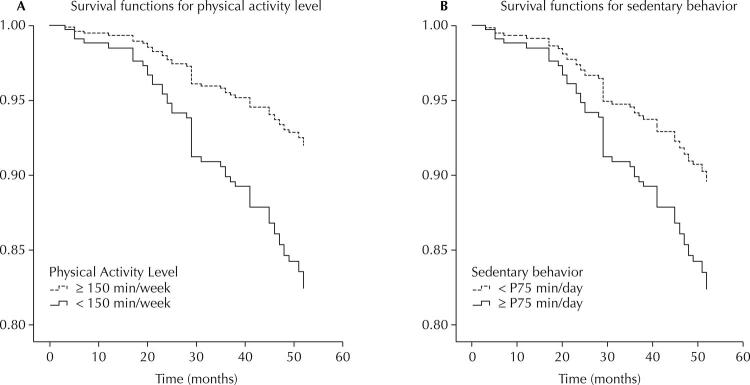
Predicted survival curves for all-cause mortality, according to the level of physical activity (A) and sedentary behavior (B).

**Figure 2:**
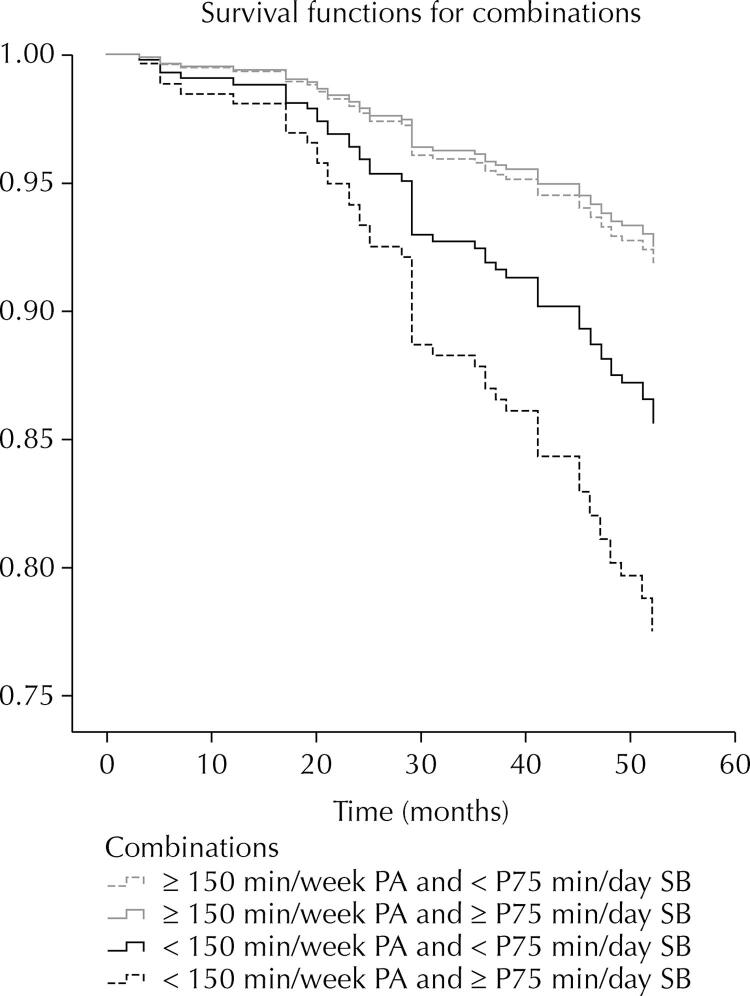
Predicted survival curves for all-cause mortality according to the combination of physical activity and sedentary behavior.

The Cox’s proportional risk regression analysis estimated the risk ratios as measures of Hazard Ratio (HR), with 95% confidence intervals (CI) for all-cause mortality, with survival time in months. The models included progressive adjustments for possible confounding factors: model 1 not adjusted; model 2 adjusted for gender and age; model 3 adjusted for gender, age, income, and number of diseases; and model 4 adjusted for gender, age, income, number of diseases, smoking and BMI, using the active segment model. Figure 1 shows the predicted survival for PA and SB and Figure 2 for combinations. The premise of proportional hazards was inspected graphically using the Kaplan–Meier graph and the log-rank (Mantel–Cox), Breslow (generalized Wilcoxon) and Tarone–Ware tests, for the variables of PA, SB and their combinations, with no violation observed.

To elucidate possible errors caused by a large number of losses at the end of the second wave of the study (n = 141), secondary analyses of proportional risks were performed considering all losses with vital living status, as a passive segment model (data not shown) and no statistical differences were observed. We adopted a significance level of 5%.

## RESULTS

A total of 332 older adults participated in the study, representing 79,2% of those eligible in the baseline study, out of which 273 were with vital living status and 59 (17.8%) died in the follow-up period, 43 of whom (72.9%) were insufficiently active (at the end of the first wave the study). Table 1 shows the distribution of descriptive variables regarding vital status. On average, those who died during the segment had lower levels of PA and longer time in SB, were older and reported worse health status, were smokers and had a lower BMI.

**Table 1:** Characteristics of participants by vital status, obtained in the first wave of the study.

	Alive		Deceased		p
	
	n	%	n	%	
Gender					0.655
Male	98	81.0	23	19.0	
Female	175	82.2	36	17.1	
Home arrangement					0.871
Without partner	142	82.6	30	17.4	
With partner	131	81.9	30	18.1	
Perception of health					0.171
Positive	109	85.8	18	14.2	
Negative	163	79.9	41	20.1	
Physical Activity					**< 0.001**
≥ 150min/week	157	90.8	16	9.2	
< 150 min/week	116	73.0	43	27.0	
Sedentary Behavior					**0.002**
< 540 min/day	211	86.1	34	13.9	
≥ 540 min/day	62	71.3	25	28.7	
Smoking history					0.269
No	245	83.1	50	16.9	
Yes	28	75.5	9	24.3	

	**Median**	**SE**	**Median**	**SE**	

Age	67.0	0.46	78.0	1.25	**< 0.001**
Number of diseases	3.0	0.16	3,0	0.39	**0.025**
Body mass index kg/m^2^	26.82	0.32	25.72	0.71	**0.021**

SE: standard error.

Figure 1 shows the survival curves of the participants according to the level of PA and SB and demonstrates higher declines in the participants with the worst behaviours.

Figure 2 shows the curves according to the combinations of PA and SB (≥ 150 min/week and < P75 min/day; ≥ 150 min/week and ≥ P75 min/day; < 150 min/week and < P75 min/day; < 150 min/week and ≥ P75 min/day).

Table 2 shows that the older adults who are insufficiently active showed, on average, a higher mortality rate than those who are sufficiently active (HR = 2.29; 95% CI = 1.18–4.44). Participants least exposed to SB (< 540 min/day) had, on average, significantly lower mortality rates than the older adults with greater exposure to SB (≥ 540 min/day) in models 1, 2, and 3. However, this effect was lost in model 4 (HR = 1.71; 95% CI = 0.97–3.02).

**Table 2:** Hazard Ratio for physical activity, sedentary behavior and physical activity combined with sedentary behaviour.

Variable	Hazard Ratio (CI 95%)							

	Model 1^a^	p	Model 2^b^	p	Model 3^c^	p	Model 4^d^	p
Physical activity		< 0.001		0.009		0.009		0.014
≥ 150min/week	1		1		1		1	
< 150 min/week	3.56 (1.94–6.54)		2.34 (1.23–4.47)		2.35 (1.23–4.49)		2.29 (1.18–4.44)	
Sedentary behaviour		**0.006**		**0.025**		**0.037**		0.063
< 540 min/day	1		1		1		1	
≥ 540 min/day	2.13 (1.24–3.65)		1.99 (1.16–3.43)		1.81 (1.03–3.17)		1.71 (0.97–3.02)	
PA and SB combinations		**< 0.001**		**< 0.001**		**< 0.001**		**< 0.001**
≥ 150 min/week e < P75 min/day	1		1		1		1	
≥ 150 min/week e ≥ P75 min/day	1.05 (0.23–4.70)		1.02 (0.22–4.56)		0.93 (0.20–4.19)		0.88 (0.19–4.01)	
< 150 min/week e < P75 min/day	2.88 (1.41–5.86)		1.78 (0.84–3.76)		1.83 (0.86–3.89)		1.81 (0.84–3.89)	
< 150 min/week e ≥ P75 min/day	4.85 (2.37–9.92)		3.36 (1.60–7.06)		3.10 (1.46–6.58)		2.92 (1.36–6.26)	

PA: physical activity; SB: sedentary behaviour; HR: hazard ratio.

^a^ Model 1 not adjusted.

^b^ Model 2 adjusted for gender and age.

^c^ Model 3 adjusted for gender, age, income and number of diseases.

^d^ Model 4 adjusted for gender, age, income, number of diseases, smoking history and body mass index.

When analysing the combinations of PA and SB, the best behaviours were used as reference (≥ 150 min/week and < P75 min/day), with no effect found in any model for the sufficiently active group with a long time in SB (≥ 150 min/week and ≥ P75 min/day) and with the group insufficiently active and with low SB (< 150 min/week and < P75 min/day) (HR = 1.81; 95% CI = 0.84–3.89). However, the group insufficiently active and with high time in SB (< 150 min/week and ≥ P75 min/day) had higher mortality rates than the reference group (HR = 2.92; 95% CI = 1.36–6.26).

## DISCUSSION

Our study aimed to examine the effects of PA and SB in isolated and combined on all-cause mortality in older adults. In the isolated PA analysis, we observed higher mortality risk rates for those who did not meet the PA recommendations. When evaluating the results for SB, people with long-term exposure to SB (≥ 540 min/day) had higher rates of risk of mortality. However, this effect was reduced when adjusting the final model for gender, age, income, number of diseases, smoking and BMI. In the analysis combinations of PA and SB, only people who did not meet the recommendations of PA and spent a long-term exposure to SB (< 150 min/week and ≥ P75 min/day) had a higher risk of mortality.

Our results showed that meeting PA recommendations reduces the risk of mortality in people with a short SB. A recent study evaluating 1,148 individuals with an average age of 70 years old, identified lower mortality risk rates in individuals with medium and high PA levels compared to individuals with low PA levels, with respectively 38% and 42% lower chances of mortality^13^.

Other studies have also been developed and found beneficial results for different levels of PA^5,6,8,25,26^. In a study that aimed to assess the benefits of PA at leisure and the risks of mortality, mortality rates were 20% lower in individuals who did not meet the recommendations of PA (0.1–< 7.5 MET h/wk) than those who did not perform PA at leisure, with stronger associations being observed as the PA level increased, reaching a threshold of 39% less chance of mortality in individuals who perform between 3 and 10 times the recommended minimum^5^.

PA accumulation is another important factor that is investigated corcerning PA levels and its health benefits. It is divided into two categories: time accumulation and accumulation of time pattern, being the first one comprinsing the total time of PA and the second one, the time pattern. This factor aims to determine which one is more important. A study that sought to evaluate the patterns of PA accumulation in 1,274 men with an average age of 78.4 years old, observed that the accumulation of 150 minutes of MVPA in sporadic minutes reduced the chance of mortality by 41% (HR = 0.59; CI = 0.43–0.81), and in men with accumulation patterns ≥ 10 minutes, no associations were observed (HR = 0.58; CI = 0.33–1.00), with the indication that the total volume and not the pattern of PA accumulation is the most important factor in mortality^8^. Another study that investigated the fragmentation of daily PA as a possible sign of physiological decline in 262 participants with an average age of 75.8 years old, found no association between the level of total PA and mortality. However, each one increased by 10% of fragmentation, a 49% increase in mortality risk was observed in the time spent on physical activities, and with each 10% increase in daily activities performed for periods < 5 minutes, a 31% higher risk of mortality was found^27^.

Considering our older adult participants, who tend to live with more NCDs, it is essential to take this factor into account when evaluating and managing it^28,29^. A study by a UK research group investigated the association between PA and all-cause mortality in more than 491,000 people with and without multimorbidity, and identified a 51% reduction in mortality in moderately active people (600 to < 3,000 MET-min/wk), and a 71% mortality reduction in those with high PA time (≥ 3,000 MET-min/wk), with both groups having multimorbidity. In participants without multimorbidity, these rates were respectively 60% and 71% in the reduction of mortality^6^, demonstrating that PA can be essential, even in individuals with high-risk factors for mortality.

When analysing the SB time in isolation, in models 1, 2 and 3 we observed higher risks of mortality for people with long time exposed to SB compared to those who spend less time exposed to this behaviour; however, this risk was mitigated in model 4 (adjusted for gender, age, income, number of diseases, smoking and BMI). This fact is controversial in the literature, as some studies have been conflicting about SB and mortality from all causes. Some studies have found associations, demonstrating that this behaviour independently can be a determining factor for mortality at high levels^8,13^; however, other studies have demonstrated that this behaviour in isolation may not be the most harmful to health^30,31^. In our study, there was also no direct association between the time exposed to SB and mortality. However, SB was shown to enhance the mortality risks in the older adults population (Table 2, model 4c).

Jefferis et al.^13^ observed a 43% increase in the risk of mortality in people exposed to SB for a long time; this percentage may increase by 17% for every additional 30 minutes of exposure to SB^8^. This confounding factor can be explained by the different evaluation methods between the studies, or by the behaviour pattern considered high, given the lack of consensus between which time exposure to SB would be harmful to the population’s health.

When analysing the combinations, we noticed that complying with recommendations of PA and reducing the time in SB demonstrate a protective function against mortality. Other studies indicate that complying with the recommendations of PA (150 to 300 min/week of MVPA) nullifies the risks of mortality, regardless of the time in SB^15,32^. A prospective cohort study involving 149,077 people, to associate sitting time and MVPA together, demonstrated that individuals who comply with the PA recommendations (150 to 299 min/week) have a lower mortality rate, regardless of time in SB, except those who are exposed to > 8 hours/day in SB. On the other hand, individuals who perform more PA than recommended (> 300 min/week) have their association with mortality eliminated, regardless of the time in SB^15^. In our study, being physically active (≥ 150 min/week) was not shown to mitigate the risks of mortality in exposed individuals ≥ 9 hours/day of SB.

A recent study carried out by a Brazilian group^33^, which investigated PA patterns, sitting time and SB breaks with 212 elderly of primary health of primary health care in Brazil, found a greater protective factor for metabolic syndrome in the most physically active group and with less time in SB. The authors also suggest that public health interventions should focus mainly on increasing PA and reducing sitting time in older adults. However, this study ignores the fact that even the most physically active individuals can spend a long time in SB, and do not include a similar group for comparison with the others, since in our study, being physically active did not nullify the mortality risks in people who spent a long time in SB.

The trained skeletal muscle has an improved ability to use fat as a substrate, being less dependent on blood glucose and muscle glycogen as substrates during PA^34^. SB has biological and underlying pathways different from physical inactivity, with concerning health risks^35^. The exacerbated time of exposure to SB can produce distinct metabolic and cardiovascular responses, thus, light-intensity PA cannot to prevent them. c.

The results of this study indicate the potentiation of the risk of death when adding the two negative behavioural situations (physical inactivity and SB). On the other hand, moderate to vigorous PA seem to mitigate the risks of early mortality, even for those older adults with prolonged exposure to SB.

The limitations of our study are present in the use of questionnaires with self-reported measures to assess PA and SB, and may present bias of forgetfulness and/or measures. This factor is minimized by the previous training of the interviewers and the evaluation of the measures only at baseline, with no changes in behaviour over time. Another limitation was the large number of people not located at the end of the second wave; however, the analyses were conducted considering this factor (data not shown) and there were no changes in the values obtained.

The strengths of our study are that it was a prospective study, with a five-year follow-up; its participants were a representative sample of a locality with small population size and [economically] predominantly on a low-income, in a developing country. Also, it has a combined analysis of PA with SB^19^.

## CONCLUSION

Our isolated analyses showed that complying with PA recommendations reduces the risk of all-cause mortality in older adults. However, the time of exposure to SB was not associated with mortality. In the combined analysis, being physically active proved to be a protective factor against the risk of mortality in people with low SB. However, a high time in SB proved to be a factor that increased the risk of mortality in insufficiently active older adults.

Such findings suggest that, in the condition of a low level of PA, a simple reduction of sitting time may be insufficient for an ideal health benefit. Public policies, such as providing specialized spaces for PA for older adult people, should encourage to regulate PA and simultaneously reduce the SB in order to prevent chronic diseases and mortality in this population.

We suggest additional studies to expand knowledge on the role of daily light physical activities in the 24-hour compositional context, and other cutoff points for SB that can ensure better quality of the behavioural guidelines of human movement.
